# Homozygous germ-line mutation of the *PMS2* mismatch repair gene: a unique case report of constitutional mismatch repair deficiency (CMMRD)

**DOI:** 10.1186/s12881-017-0391-x

**Published:** 2017-04-05

**Authors:** N. C. Ramchander, N. A. J. Ryan, E. J. Crosbie, D. G. Evans

**Affiliations:** 1 The University of Manchester, Oxford Road, Manchester, M13 9PT UK; 2 Clinical Research Fellow, Division of Molecular & Clinical Cancer Sciences, Faculty of Biology, Medicine and Health, Fifth Floor - Research, St Mary’s Hospital, University of Manchester, Oxford Road, Manchester, M13 9WL UK; 3 Gynaecological Oncology, Division of Molecular & Clinical Cancer Sciences, Faculty of Biology, Medicine and Health, Fifth Floor - Research, St Mary’s Hospital, University of Manchester, Oxford Road, Manchester, M13 9WL UK; 4 Department of Obstetrics and Gynaecology, Manchester, Academic Health Science Centre, Central Manchester University Hospitals NHS Foundation Trust, Manchester, UK; 5 Medical Genetics and Cancer Epidemiology, Genomic Medicine, Manchester Academic Health Science Centre, The University of Manchester, Oxford Road, Manchester, M13 9WL UK; 6 Central Manchester University Hospitals NHS Foundation Trust, Saint Mary’s Hospital, Oxford Road, Manchester, M13 9WL UK

**Keywords:** Constitutional mismatch repair deficiency, CMMRD, Mismatch repair, MMR, PMS2, Lynch syndrome, Turcot syndrome

## Abstract

**Background:**

Constitutional mismatch repair deficiency syndrome results from bi-allelic inheritance of mutations affecting the key DNA mismatch repair genes: *MLH1*, *MSH2*, *MSH6* or *PMS2.* Individuals with bi-allelic mutations have a dysfunctional mismatch repair system from birth; as a result, constitutional mismatch repair deficiency syndrome is characterised by early onset malignancies. Fewer than 150 cases have been reported in the literature over the past 20 years. This is the first report of the founder *PMS2* mutation - NM_000535.5:c.1500del (p.Val501TrpfsTer94) in exon 11 and its associated cancers in this family.

**Case presentation:**

The proband is 30 years old and is alive today. She is of Pakistani ethnic origin and a product of consanguinity. She initially presented aged 24 with painless bleeding per-rectum from colorectal polyps and was referred to clinical genetics. Clinical examination revealed two* café-au-lait* lesions, lichen planus, and a dermoid cyst. Her sister had been diagnosed in childhood with an aggressive brain tumour followed by colorectal cancer. During follow up, the proband developed 37 colorectal adenomatous polyps, synchronous ovarian and endometrial adenocarcinomas, and ultimately a metachronous gastric adenocarcinoma. DNA sequencing of peripheral lymphocytes revealed a bi-allelic inheritance of the *PMS2* mutation NM_000535.5:c.1500del (p.Val501TrpfsTer94) in exon 11. Ovarian tumour tissue demonstrated low microsatellite instability. To date, she has had a total abdominal hysterectomy, bilateral salpingo-oophorectomy, and a total gastrectomy. Aspirin and oestrogen-only hormone replacement therapy provide some chemoprophylaxis and manage postmenopausal symptoms, respectively. An 18-monthly colonoscopy surveillance programme has led to the excision of three high-grade dysplastic colorectal tubular adenomatous polyps. The proband’s family pedigree displays multiple relatives with cancers including a likely case of ‘true’ Turcot syndrome.

**Conclusions:**

Constitutional mismatch repair deficiency syndrome should be considered in patients who present with early onset cancer, a strong family history of cancer, and cutaneous features resembling neurofibromatosis type I. Immunohistochemistry analysis of tumour and normal tissue is sensitive and specific for identifying patients with mismatch repair deficiency and should direct DNA sequencing of lymphocytic tissue to establish a diagnosis. Microsatellite instability status appears to be of little value in identifying patients who may have constitutional mismatch repair deficiency syndrome.

## Background

Mismatch repair (MMR) proteins maintain genomic stability [1]. Four of these proteins are pivotal to DNA mismatch repair: MLH1, MSH2, MSH6 and PMS2. Mismatch repair involves the recognition and correction of single DNA base-base mismatches and insertion-deletion loops (IDL’s) that arise during cellular replication, preventing these mutations from persisting in daughter cells [2].

Lynch syndrome (formerly known as hereditary nonpolyposis colorectal cancer or HNPCC) is an autosomal dominant cancer predisposition syndrome with a variable penetrance; it results from the inheritance of a heterozygous mutation affecting one of the key mismatch repair genes: *MLH1, MSH2, MSH6*, or *PMS2* [3–5]*.* The loss of the wild-type allele leads to MMR deficiency, and a hyper-mutated phenotype. The repetitive coding sequences of microsatellites are particularly susceptible to acquiring insertions and deletions of nucleotide bases during cellular replication, which result in frameshift mutations. Mismatch repair deficiency promotes the persistence of these frameshift mutations in daughter cells, measured as microsatellite instability (MSI). Microsatellite instability-high (MSI-H) tumours are characteristic of Lynch syndrome associated colorectal cancer (CRC) [6]. A severe variant of Lynch syndrome is constitutional mismatch repair deficiency (CMMRD), which results from bi-allelic inheritance of an MMR gene mutation [7, 8]. With it comes a poor prognosis; over 50% of patients develop malignant brain tumours, over 30% develop haematological malignancies, and 40% develop digestive tract tumours, all at very young ages [9]. The most frequent cancers observed in CMMRD are brain gliomas, non-Hodgkin’s lymphomas and CRC’s, with an average age at diagnosis of 9.5 years, 5 years, and 16 years, respectively [10]. The cancer spectrum in CMMRD appears to be related to the gene mutated; homozygous carriers of *MSH6* and *PMS2* mutations develop brain tumours in the first decade of life [10]. Importantly, over 40% of individuals homozygous for a *PMS2* mutation develop second primary malignancies. This is in contrast to the 22% of individuals homozygous for *MLH1/MSH2* mutations that develop second primary malignancies [10]. This difference may be because individuals with homozygous *PMS2* mutations often survive their first malignancy, unlike their *MLH1*/*MSH2* counterparts who develop aggressive haematological malignancies at an average age of 2.5 years. *Café-au-lait* (CAL) lesions are the most common dermatological manifestation of individuals with CMMRD [11]. Consanguinity of the parents and/or homozygosity for a founder mutation is observed in over 50% of reported cases of CMMRD.

## Case presentation

**Fig. 1:**
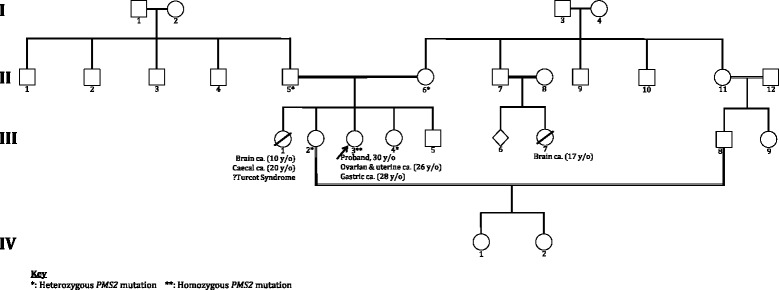
Family pedigree for the familial *PMS2* mutation NM_000535.5:c.1500del (p.Val501TrpfsTer94) in exon 11, of a family of Pakistani ethnic origin living in the UK. The proband (*III:3,* arrow)*,* is homozygous for this *PMS2* mutation. She presented with café-au-lait lesions, lichen planus, a dermoid cyst, and bleeding per-rectum. She has developed 37 benign colorectal adenomatous polyps to date. She has developed ovarian and endometrial cancer (both at age 26) and gastric cancer (age 28). *III:1* developed a parietal lobe astrocytoma (age 10) and a caecal adenocarcinoma of which she died (age 20); her mutation status is unknown, however *III:1’s* history is representative of true Turcot syndrome. *III:9* died of a brain tumour (age 17); her mutation status is unknown. *III:5* has declined genetic testing for the time being. II:5 and II:6, *II:7* and *II:8*, *II:11* and *II:12* are distant cousins

**Fig. 2:**
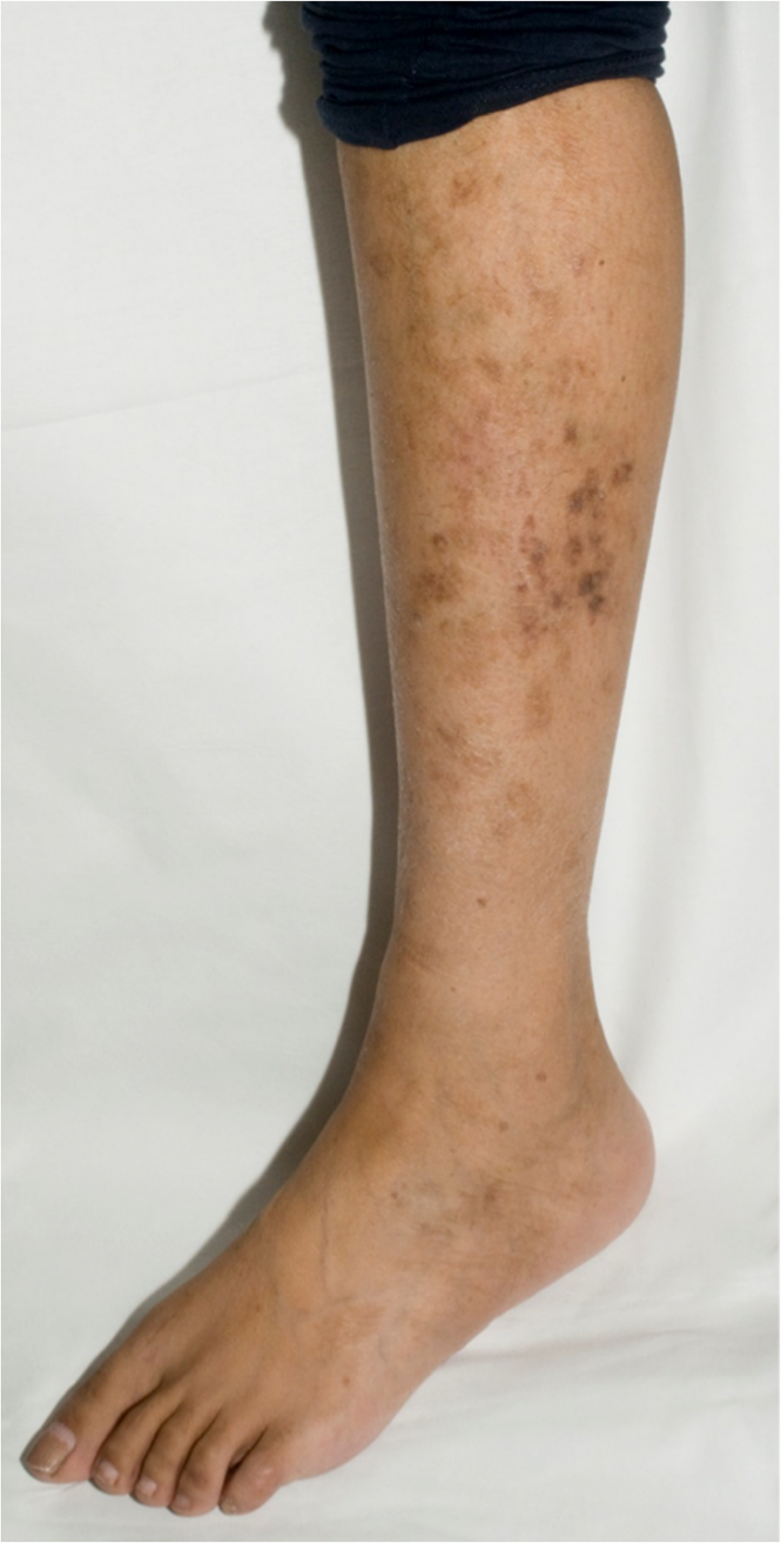
Photograph of the proband’s left leg showing café-au-lait lesions on a background of lichen planus

The proband is a 30-year-old female of Pakistani ethnic origin and a product of consanguinity; her parents are distant cousins. Her family pedigree is depicted in Fig. 1. The prominent feature was that a sister *(III:1)* had developed a high-grade parietal lobe astrocytoma aged 10 years but died from a metastatic primary adenocarcinoma of the caecum aged 20 years. The proband *(III:3)* presented aged 24 years, with painless bleeding per-rectum (PR) and iron deficiency anaemia. This was investigated by colonoscopy at which 10 colonic tubullo-villous adenomatous polyps with low-grade dysplasia were excised. Further clinical investigation was prompted by her unusual presentation. On examination, she had two distinct CAL lesions, one on her right arm and one on her left leg. Both CAL lesions had irregular edges with foci of hypopigmentation within areas of hyperpigmentation (Fig. 2). She also had widespread lichen planus mostly concentrated to her lower limbs, and a dermoid cyst on her left lower back.

Initial investigations were for *MUTYH* associated polyposis (MAP) and Lynch syndrome given the early age of onset of polyps, dermatological manifestations, and very early onset family history of CRC. Direct sequencing of lymphocytic tissue for *MUTYH* did not reveal any mutations. All tissue analyses performed are summarised in Table 1. Initial immunohistochemistry (IHC) analysis was deemed inconclusive for Lynch syndrome due to perceived poor technical quality of PMS2 staining; there was universal absence of PMS2 antibody uptake in tumour and control tissue.

**Table 1:** Molecular analysis of polypoid and cancerous tumour tissue resected from the proband

Mismatch repair protein immunohistochemistry	
Colorectal polypoid tissue	• >80% positivity for MLH1, MSH2 and MSH6 • Inconclusive result for PMS2 (reported poor technical quality of staining)
Ovarian tumour tissue	• >80% positivity for MLH1, MSH2, MSH6 • 0% positivity for PMS2. In addition, there was 0% positivity for PMS2 expression in control tissue
Cancer tumour characteristics	
Ovarian tumour tissue	• Positive for CK7, PAX-8 and ER • MSI low
Gastric tumour tissue	• Positive for CK7, CDX2 with focal positivity for CX20 • Negative expression of ER, PR, WT1, PAX8 and GCDFP15 • There was no evidence of loss of E-cadherin expression

Molecular characteristics of the colorectal polypoid tissue, ovarian cancer tumour tissue, and gastric cancer tumour tissue resected from the proband. Initial colorectal polypoid tissue analysis was deemed inconclusive due to perceived poor technical quality of the staining due to universally absent uptake of the PMS2 antibody

**Fig. 3:**
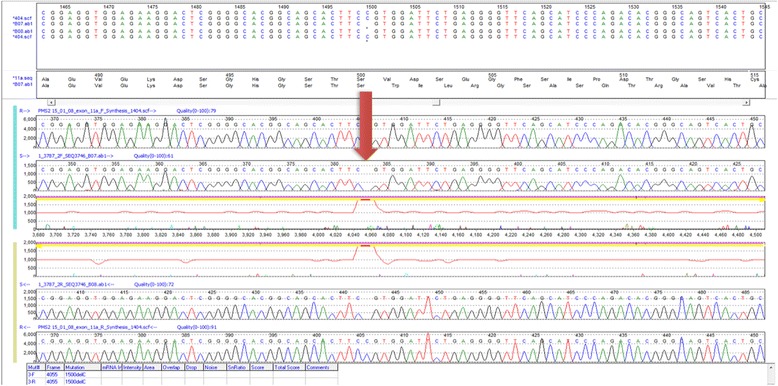
Sanger sequence of the proband’s peripheral lymphocytes showing *PMS2* mutation NM_000535.5:c.1500del (p.Val501TrpfsTer94) in exon 11. Arrow points to homozygous loss of cytosine at the 1500 frame

A pelvic ultrasound scan was performed later that year when the patient reported symptoms of bloating. This revealed malignant-looking bilateral ovarian cysts. Bilateral ovarian cystectomy revealed grade 1/grade 2 endometrioid adenocarcinomas affecting both ovaries. Peritoneal washings demonstrated the presence of adenocarcinoma cells. The proband’s tumour phenotype is summarised in Table 2. Immunohistochemistry was repeated on ovarian tissue; 0% tumour positivity and 0% control positivity for PMS2 was established. Microsatellite instability analysis demonstrated the ovarian tumour to be microsatellite instability-low (MSI-L). Sanger dye terminator sequencing of lymphocytic tissue identified that the proband was homozygous for the *PMS2* founder mutation NM_000535.5:c.1500del (p.Val501TrpfsTer94) in exon 11, and a diagnosis of CMMRD was made (Fig. 3). The described *PMS2* mutation NM_000535.5:c.1500del (p.Val501TrpfsTer94) leads to a downstream stop codon at the 94^th^ amino acid, creating a truncated protein. Given the lack of protein expression on IHC, it is likely that truncation leads to nonsense mediated decay with no protein product. The variant has been seen twice on the Exome Aggregation Consortium (ExAc) with a frequency of 0.0000165, which represents 2 of 30,610 individuals tested.

**Table 2:** Cancer tumour tissue histology and the management of malignancies in the proband

Year	Proband’s age	Location	Tumour histology	Stage (Grade)	Management
Aug. 2012	26y9m	Ovaries	Endometrioid adenocarcinoma	FIGO Stage 1c (Grade 2)	Total abdominal hysterectomy with bilateral salpingo-oophorectomy
Oct. 2012	26y10m	Uterus	Endometrioid adenocarcinoma	FIGO 2009 Stage 1a (Grade 1)	Total abdominal hysterectomy with bilateral salpingo-oophorectomy
Dec. 2013	28y0m	Stomach	Gastric Adenocarcinoma	T3N2M0 (Grade 3)	Total gastrectomy with neoadjuvant chemotherapy

The proband has developed multiple malignancies since first presenting at age 24. Her first malignancy (ovarian cancer) was diagnosed less than three years after initial presentation. Despite presenting with bleeding PR, the patient has not yet developed colorectal cancer. Aggressive surgical management was used for her gynaecological malignancies, and a combination of neoadjuvant chemotherapy and surgery was used for her gastric malignancy

The proband underwent full staging surgery with a total abdominal hysterectomy (TAH), bilateral salpingo-oophorectomy (BSO), and partial omentectomy. No residual ovarian disease was present, which corresponds to an International Federation of Gynecology and Obstetrics FIGO Stage 1c (Grade 2) endometrioid adenocarcinoma of the ovary. An incidental, synchronous FIGO Stage 1a (Grade 1) endometrioid adenocarcinoma of the endometrium was identified during routine post operative histological examination of the uterus. Adjuvant chemotherapy was not used.

The proband was then initiated on an 18-monthly colonoscopy-surveillance program and advised to take daily aspirin (75mg-150mg) for chemoprevention against MSI-H CRC. In total, the proband has had 37 adenomatous large bowel polyps excised; three of these were high-grade dysplastic tubular adenomatous polyps of the rectosigmoid that were identified and excised as part of her surveillance programme following diagnosis. She was also initiated on oestrogen-only hormone replacement therapy (HRT) following unsuccessful vasomotor menopausal treatment with venlafaxine.

The following year she developed nausea and vomiting, and as a result, an urgent oesophago-gastro-duodenoscopy was performed. Gastric biopsy revealed a poorly differentiated adenocarcinoma in the body of the stomach and confirmed it was a primary tumour that did not represent recurrence (Table 1). She was treated with neoadjuvant chemotherapy followed by total gastrectomy and completion of omentectomy, with the excision of 0.5 cm of the lower oesophagus and 1.3 cm of the duodenum. This revealed a poorly differentiated primary gastric adenocarcinoma (T3N2M0) with diffuse widespread infiltration through the *muscularis propria* into the subserosal tissue. Four lymph nodes showed evidence of metastatic adenocarcinoma.

Immunohistochemistry analysis for MLH1, MSH2, MSH6 and PMS2 was performed on 4μm formalin fixed, paraffin embedded tissue sections using the automated Ventana BenchMark ULTRA IHC/ISH Staining Module (Ventana Co., Tucson, AZ, United States) together with the OptiView, 3′ diaminobenzidine (DAB) version 5 detection system (Ventana Co., Tucson, AZ, United States). 0% staining intensity for an MMR protein was regarded as negative expression of that protein.

Microsatellite instability analysis was performed on ovarian tumour tissue using the Promega MSI analysis system v1.2 (Wisconsin, United States). The MSI Analysis System includes fluorescently labelled primers for co-amplification of seven markers including five mononucleotide repeat markers (BAT-25, BAT-26, NR-21, NR-24 and MONO-27) and two pentanucleotide repeat markers (Penta C and Penta D). The mononucleotide markers were used for MSI determination, and the pentanucleotide markers were used to detect potential sample mix-ups and/or contamination. In addition, control lymphocytic tissue from the proband was analysed using the same platform.

Peripheral lymphocytes were utilised for genetic sequencing. Mutations in the *MUTYH* (formerly known as *MYH*) and MMR genes were searched for using dye terminator Sanger sequencing on ABI Genetic Analysis of the exon regions of the coding sequences. Sequence analysis of exons 2, 4, 6, 7, 13 and 14 of the *MUTYH* gene was used to detect common Indian and Pakistani mutations (p.Tyr104Ter and p.Glu480Ter) and common European mutations (p.Tyr179Cys and p.Glu396Asp). The reference sequence for the *MUTYH* gene was NM_001128425.1. All 19 exons in *MLH1*, 16 exons in *MSH2* and 10 exons in *MSH6* were sequenced in both directions. Analysis of mutations in the *PMS2* exon coding sequence was complicated by presence of multiple pseudogenes corresponding to exons 1–5 of *PMS2* and a single large pseudogene encompassing exons 9 and 11–15 of *PMS2*. Exon 11 particularly had a high homology to the pseudogene. Long range PCR was performed first to amplify *PMS2* (and not the pseudogene *PMS2CL*) prior to Sanger sequencing. Multiple ligation dependent probe amplification (MLPA) was used to detect dosage abnormalities in exons 1–11 of *PMS2* using kit P008-B1 [MRC-Holland]. Due to gene conversion and technical issues, analysis of exons 13–15 was not performed. The reference sequence for *PMS2* was NM_000535.5.

Tissue analysis results are summarised in Table 1. Of note, the initial IHC staining of polypoid tissue and non-malignant control tissue was deemed inconclusive due to absent uptake of the PMS2 antibody in both the polypoid tissue and control tissue; this was attributed to poor technical quality of the staining. This delayed diagnosis until subsequent IHC analysis of the ovarian tumour, which established 0% immunopositivity for PMS2 in tumour tissue and control tissue. This prompted DNA sequencing of lymphocytic tissue, resulting in diagnosis of the proband as homozygous for the founder *PMS2* mutation NM_000535.5:c.1500del (p.Val501TrpfsTer94) in exon 11.

## Discussion

Here we present the rare case of a young woman with CMMRD, a severe variant of Lynch syndrome. The proband presented in her twenties with PR bleeding, went on to develop multiple gynaecological malignancies, and ultimately, an advanced primary gastric tumour. Both of these cancer types are rare amongst individuals with CMMRD; the later represents the first reported gastric malignancy in a confirmed case of CMMRD. CMMRD is rare and poses both a diagnostic and managerial challenge. It is interesting to note the delay in onset of disease in this individual. One may speculate that this results from retained PMS2 function despite the mutation. The complete loss of protein expression on IHC suggests that this explanation is unlikely. In addition, pathological mutations identified in distal exons indicate the structural functionality of *PMS2* lies beyond exon 11. Both of these observations would support nonsense decay of *PMS2* consequent to the reported mutation.

A review of 146 cases of CMMRD conducted by Wimmer et al. concluded that *PMS2* mutations were responsible for 60% of cases, with the remaining being *MLH1*, *MSH2* and *MSH6* mutations; this is in contrast to Lynch syndrome, where heterozygous *MLH1* and *MSH2* mutations are responsible for the majority of cases [10]. In CMMRD, *MLH1*/*MSH2* mutations carry a worse prognosis compared to *MSH6*/*PMS2* mutations; individuals with *MSH6* or *PMS2* mutations often survive their first malignancy and develop secondary malignancies, as was the case in the proband. Heterozygous *PMS2* mutations appear to display an attenuated Lynch syndrome phenotype. This is reflected not only by the later age of onset of cancer, but also by the lower penetrance of cancer [12]. A potential explanation for this could be the partial compensation of absent PMS2 by MLH3, which can form a functional heterodimeric protein with MLH1 that has mismatch repair capacity [5]. Neither of the proband’s parents have a history of cancer, reflecting this attenuated phenotype. This presents a diagnostic challenge and highlights the difficulty in identifying familial *PMS2* mutations. Several features in the history alluded to a severe inherited cancer predisposition. This included the family history of childhood malignancies, parental consanguinity, early presentation of the patient and the presence of cutaneous signs resembling neurofibromatosis type I (NF-1). The identification of the proband’s homozygous *PMS2* mutation status has led to the diagnosis of several family members with Lynch syndrome (Fig. 1).

Bakry et al. report that immunohistochemistry of tumour tissue and intratumoural normal tissue is 100% sensitive in detecting MMR deficiency resulting from bi-allelic gene inactivation in CMMRD [8]. Therefore, in cases of early onset cancer, with a history of consanguinity and cutaneous features resembling NF-1, Durno et al. propose performing IHC for MMR proteins on tumour and normal tissue; abnormal IHC in both tumour and normal tissue should alert to CMMRD, and relevant DNA sequencing should follow [11]. Microsatellite instability is the molecular hallmark of Lynch syndrome associated tumours, and hence MSI status is part of the revised Bethesda guidelines for identifying Lynch syndrome [6, 13]. Microsatellite instability status in CMMRD, however, has been reported as neither sensitive nor specific; particularly in extra-intestinal tumours [8]. This suggests it is less reliable as an assessment tool for CMMRD than it is for Lynch syndrome. In the proband, therefore, the fact that her ovarian tumour was MSI-L did not prevent escalation of investigation for MMR deficiency to include genetic sequencing.

Another diagnostic challenge arises from the presentation individuals with CMMRD: often with CAL lesions, which raises the differential of NF-1. Neurofibromatosis type I is an autosomal dominant condition, and a clinical diagnosis can be made using the National Institute of Health (NIH) criteria [14]. Whilst other features of NF-1 are observed in patients with CMMRD, such as Lisch nodules, plexiform neurofibromas and axillary freckling, they rarely meet the diagnostic criteria [15]. The proband did not meet these criteria. The CAL lesions observed in the proband displayed diffuse borders with areas of hypopigmentation within lesions of hyperpigmentation, as reported elsewhere in the CMMRD literature [10]. This is in contrast to the homogenous, well-demarcated CAL lesions observed in NF-1 [14, 16, 17].

Looking to other reported cases of CMMRD, brain tumours have been observed in 53% of cases [9]. Gliomas are the most common, and present in the first decade of life. Other brain tumours include supratentorial neuroectodermal tumours and meduloblastomas. *III:1* developed a parietal lobe astrocytoma aged 10 followed by CRC aged 20. Genetic testing was never performed, however her history strongly reflects *PMS2* mutation homozygosity. It is interesting to note that *III:1*’s history could be representative of ‘true’ Turcot syndrome. Brain tumour polyposis syndrome (BTPS) is defined by a combination of synchronous or metasynchronous brain and colonic neoplasms [18–20]. BTPS is divided into type 1 and type 2. The distinctions between BTPS type 1 and BTPS type 2 lie in their modes of inheritance and tumour phenotypes. BTPS type 1 refers to autosomal recessive inheritance of MMR gene mutations with the development of Lynch syndrome and the childhood (<20 years) onset of brain glial tumours (glioblastomas or astrocytomas). BTPS type 1 is in keeping with McKusick’s original description of Turcot syndrome and with a CMMRD spectrum disease [21, 22]. BTPS type 2 refers to autosomal dominant inheritance of APC gene mutations with the development of Familial Adenomatous Polyposis (FAP), and the development of brain medulloblastomas [20]. The authors suggest that a diagnosis of Turcot syndrome be restricted to patients with homozygous loss of MMR, that is CMMRD, and not in those with heterozygous loss of MMR or FAP with neurological tumours.

The management of CMMRD individuals is without a solid evidence base. The effectiveness of chemotherapy in patients with MSI-H Lynch syndrome associated tumours is unknown; there is some evidence that these tumours are less responsive to 5-fluorouracil based chemotherapy [23, 24]. Importantly, there is also the possibility of accelerated mutagenesis and the development of second malignancies when CMMRD cells are exposed to alkylating agents [25]. Our patient was not treated with adjuvant chemotherapy. This was in light of the uncertain benefit of such treatment in CMMRD and the risk of accelerated tumourigenesis. The patient did however receive neoadjuvant chemotherapy preceding her gastrectomy.

Another important aspect of the proband’s management was the decision to start her on oestrogen-only HRT. This was to reduce the symptoms of menopause, as well as protect against the development of CRC and osteoporosis. The proband was also advised to take daily aspirin. This has been shown to reduce the incidence of CRC in Lynch syndrome [26]. Further clarity is required regarding its efficacy in CMMRD patients, as the molecular mechanism of aspirin in preventing colorectal cancer is attributed to its suppressive effect of an MSI phenotype, via a nitric oxide mediated genetic selection that enhanced apoptosis of cells undergoing MSI [27].

A wide tumour phenotype is observed in CMMRD as well as a variable age of tumour onset. The age-dependent levels of mitotic activity in local tissue could explain this. Tissue with increased mitotic activity, combined with an inability to correct accumulating DNA mismatches and IDL’s, is predisposed to tumourigenesis. This wide tumour spectrum complicates the implementation of an effective, comprehensive surveillance program. Screening should target the most common cancers observed in CMMRD patients for that age group. The European Consortium provided screening guidance for the management of individuals with CMMRD [9]. The routine colonoscopy surveillance of patients with Lynch syndrome has reduced CRC-mortality by over 60% [28]. To improve sensitivity, it is recommended to combine chromoscopy with colonoscopy [29, 30]. The European Consortium recommend annual video-capsule endoscopy (VCE) and ileocolonoscopy for patients with CMMRD to identify small and large bowel cancer [9]. Patients with CMMRD who have multiple colorectal adenomas are recommended a shorter screening interval of 6 months. The proband is currently on an 18-monthly colonoscopy and VCE-screening program, in keeping with the standard UK Lynch syndrome protocol [31]. An annual or 6-monthly colonoscopy program was not utilised due to the absence of a robust evidence base and the difficulty of the bowel prep following her gastrectomy. Her surveillance program has resulted in the identification and excision of three adenomatous polyps with high-grade dysplasia, and so in the proband, surveillance has been successful.

Psychosocial support is central to providing holistic care for patients, as well as the families of patients, with CMMRD. This can improve familial uptake of diagnostic testing and compliance with surveillance programs [32]. The use of a multidisciplinary team throughout the proband and her family’s care has therefore been, and continues to be, vital.

## Conclusions

In conclusion, the proband is part of the novel CMMRD cohort described over the last 20 years; we describe here for the first time her family’s *PMS2* mutation. She is unique within this cohort in that she did not develop a childhood malignancy, but rather presented in the third decade of her life with rectal bleeding. Even more interesting is that further investigation did not reveal cancer. Her primary tumours were bilateral ovarian endometrioid adenocarcinomas. Her gastric malignancy was a metachronous tumour unrelated to her endometrial and ovarian tumours and represents the first of its kind in the CMMRD literature. Constitutional mismatch repair deficiency is a poorly described cancer predisposition syndrome. We have outlined the diagnosis and treatment of this case, which highlights the complexity of diagnosing CMMRD. The proposed guidelines by Durno et al. for identifying patients at risk of having CMMRD, along with the European consortium’s proposed guidelines on surveillance in CMMRD, are therefore invaluable resources and should be used to direct the diagnosis and management of individuals with CMMRD [9, 11].

## Abbreviations

BSO: Bilateral salpingo-oophorectomy
BTPS: Brain tumour polyposis syndrome
CAL: Café-au-lait lesion
CMMRD: Constitutional mismatch repair deficiency
CRC: Colorectal cancer
FIGO: International Federation of Gynaecology Oncology
HRT: Hormone replacement therapy
IDL: Insertion-deletion loop
IHC: Immunohistochemistry
MAP: MUTYH associated polyposis
MMR: Mismatch repair
MSI: Microsatellite instability
MSI-H: Microsatellite instability-high
MSI-L: Microsatellite instability-low
NF-1: Neurofibromatosis type 1
PR: Per-rectum
TAH: Total abdominal hysterectomy
VCE: Video capsule endoscopy
